# Interaction of apelin, elabela and nitric oxide in schizophrenia patients

**DOI:** 10.2478/jomb-2019-0029

**Published:** 2020-01-23

**Authors:** Zekiye Catak, Hilal Kaya, Esra Kocdemir, Kader Ugur, Guzel Saadet Pilten, Meltem Yardim, Ibrahim Sahin, Agirbas Esra Piril, Suleyman Aydin

**Affiliations:** 1 Health Sciences University, Elazig Fethi Sekin City Hospital, Medical Biochemistry, Elazig, Turkey; 2 Elazig Mental Health Hospital, Psychiatrist, Elazig, Turkey; 3 Kovancilar State Hospital, Medical Biochemistry, Elazig, Turkey; 4 Firat University Medical School, Department of Internal Medicine (Endocrinology and Metabolism Diseases), Elazig, Turkey; 5 Health Sciences University, Bagcilar Training and Research Hospital, Medical Biochemistry, Bagcilar/Istanbul, Elazig, Turkey; 6 Firat University Medical School, (Firat Hormones Research Group), Medical Biochemistry, Elazig, Turkey; 7 Firat University Medical School, (Firat Hormones Research Group), Medical Biochemistry, Elazig, Turkey + Erzincan Binali Yildirim University Medical School, Medical Biology, Erzincan, Turkey; 8 Firat University Medical School (Medical School Student), Elazig, Turkey

**Keywords:** schizophrenia, elabela, apelin, nitric oxide, azot oksid, apelin, elabela, šizofrenija

## Abstract

**Background:**

Apelin (APLN), elabela (ELA), and nitric oxide (NO) have effects on physiological and behavioural properties in biological systems. This study was designed to determine APLN, ELA and NO levels in schizophrenia patients and assess whether these molecules are of diagnostic value.

**Methods:**

A total of 33 schizophrenic patients and 32 ageand sex-adjusted healthy participants were included in the study. ELA, APLN and NO levels were measured using ELISA methods.

**Results:**

Although the ELA and NO levels of the patients were lower than the control group, APLN levels were higher (p = 0.039, p = 0.019, p = 0.048, respectively). There was a significant negative correlation between APLN levels and triglyceride (TG) and body mass index (BMI) levels (r = -0.426, p = < 0.001 and r = -0.330, p = 0.007, respectively). Respectively, the areas under the receiver-operating characteristic (ROC) curves of the ELA/APLN, ELA/NO and APLN/NO ratios were 0.628, 0.590 and 0.709, 95% confident intervals (CI): 0.491-0.764, 0.450-0.730 and 0.579-0.840.

**Conclusions:**

Decreased levels of ELA and NO and increased APLN levels in schizophrenia suggest that these molecules may be involved in its etiopathology. The APLN/NO ratio also seems to show promise in the diagnosis of the disease and may be used in future.

## Abstract (sr)

**Uvod:**

Apelin (APLN), elabela (ELA), i azot oksid (NO) imaju efekte na fiziološke i bihevioralne osobine u biološkim sistemima. Ova studija je postavljena tako da odredi nivo APLN, ELA i NO kod pacijenata sa šizofrenijom i da proceni da li su ovi molekuli imaju dijagnostičku vrednost.

**Metode:**

U studiju je uključeno ukupno 33 bolesnika sa šizofrenijom i 32 zdrava učesnika prilagođene starosti i pola. Nivoi ELA, APLN i NO su mereni korišćenjem ELISA metoda.

**Rezultati:**

Iako su nivoi ELA i NO kod pacijenata bili niži nego kod kontrolne grupe, nivoi APLN bili su viši (p = 0,039, p = 0,019, p = 0,048). Došlo je do značajne negativne korelacije između nivoa APLN i nivoa triglicerida (TG) i indeksa telesne mase (BMI) (r = -0,426, p = < 0,001 i r = -0,330, p = 0,007, respektivno). Površine ispod ROC krive za ELA/APLN, ELA/NO i APLN/NO su bile 0,628, 0,590 i 0,709, respektivno, 95% intervali poverenja (CI): 0,491-0,764, 0,450-0,730 i 0,579-0,840.

**Zaključak:**

Smanjeni nivoi ELA i NO i povećani nivo APLN kod šizofrenije ukazuju da ovi molekuli mogu biti u vezi sa njenom etiopatologijom. Izgleda da će odnos APLN/NO moći da se koristi u dijagnostici bolesti u budućnosti.

## Introduction

Schizophrenia is a complex psychiatric disease characterised by delusions and hallucinations in which patients suffer from severe disabilities, including impairment of speech, behaviour and reality monitoring, and often leads to an early death [1] [2]. Despite advances in antipsychotic treatments, it continues to be a major psychotic disorder [3]. This disease affecting ∼1% of the society is a cause of significant burden on patients, caregivers and also the economy of the country [2] [4]. In 2013, it was estimated that the economic burden of schizophrenia in the United States was $156 billion [2].

Elabela (ELA) is a peptide of 32 amino acids that activates the apelin receptor (APJ) [5] [6]. It plays a role in various biological events such as self-renewing human embryonic stem cells in embryonic and adult periods, endoderm differentiation, cardiac morphogenesis, bone formation, regulation of blood pressure, water and food intake [7]. ELA appears in many tissues including the APJ such as placenta, heart, kidney, lung, liver, brain, skeletal muscle, gastrointestinal system [8]. Before ELA was discovered, it was thought that apelin (APLN) was the only ligand of the G protein-dependent APJ [8].

APLN is an endogenous peptide that is widely expressed in the human body. It is involved in central hypothalamic regulation [9] [10]. Due to the localisation of APLN and APJ in the limbic structure, it was also thought to have a potential healing role in stressinduced emotional response [11]. In an animal study, central administration of APLN induced depressive behaviour [12]. Intra-cerebrovascular injection of APLN was also reported to induce an antidepressantlike effect by activating phosphatidylinositol 3-kinase (PI3K) and extracellular signal-regulated kinases 1 and 2 (ERK1/2) signalling pathways in stressed rats, and aid recognition memory [13]. In children with attention deficit and hyperactivity disorder, plasma APLN levels were significantly higher in boys than in the control group [14]. Furthermore, APLN levels in patients with autistic spectrum disorder were reduced considerably [15]. How APLN affects these neural mechanisms has not been fully explained.

However, APLN shows these effects are at least partially by activating the L-arginine/nitric oxide synthase/nitric oxide pathway (NOS, NO) [16] [17]. NO is also associated with several mood disorders [18]. For example, increased arginase activity in major depression decreases NO synthesis and affects depression symptoms [19]. From an assessment of blood NO levels of patients with schizophrenia, no consensus has been established, with some studies suggesting lower levels, whereas others found higher levels [18] [20] [21]. However, peripheral NO metabolites may have significant effects on the NO changes within the central nervous system [18].

To our knowledge, no studies have investigated the relationship between APLN, ELA and schizophrenia, although NO levels have been studied in schizophrenia patients. Therefore, we aimed to determine how APLN, ELA and NO levels change in schizophrenia patients.

## Materials and Methods

We included patients diagnosed with schizophrenia based on the Structured Clinical Interview for DSM-IV (SCID) criteria which applied to the Mental Health and Diseases Hospital in Elazig, Turkey. The control group consisted of 32 age-and gendermatched volunteers who were confirmed to be healthy by physical examination and laboratory tests.

The study protocol was explained to the relatives of all participants by a psychiatry specialist through the use of appropriate language and explanations, ensuring that all steps of the study were fully understood. Written informed consent forms were obtained from all patients' relatives and control group participants. The study was approved by the non-invasive research ethics committee of Fırat University Faculty of Medicine (decision date: 16.11.2017, decision no: 25).

Participants in the control group were excluded if they had a history of abuse of illicit substances, a history of familial or individual mental illness, or a history of medical drug use in the last 3 months. Participants had to have no history of substance dependence other than smoking. Patients with type 1 or type 2 diabetes, cancer, other infectious diseases, or other chronic diseases such as cardiovascular disease, were also excluded. The schizophrenia group included 33 patients with a history of 5 + years of schizophrenia who were treated with oral antipsychotic medication in stable doses and had no other chronic disease. The mean duration of disease was 17.5 ± 9.5 years in the patient group. Antipsychotic drugs used were clozapine [4], risperidone [13], haloperidol [4], sulpiride [6], quetiapine [16], olanzapine [11], lorazepam [4], and biperiden [10].

### Laboratory Analyses

Fasting blood samples from patients and healthy controls were taken from the antecubital vein in the sitting position. It was centrifuged at 3500 rpm for 5 min. All serum samples were aliquoted into 2 Eppendorf tubes for routine biochemical and ELISA analysis.

Serum fasting glucose, low-density lipoprotein cholesterol (LDL-C), high-density lipoprotein cholesterol (HDL-C), total cholesterol (TC) and triglyceride (TG) levels were measured without delay in the routine laboratory of Elazig Mental Hospital (Cobas 6000, Roche Hitachi, Tokyo, Japan). Serum aliquots used for APLN, ELA, and NO measurements were stored at -20 °C until analysed with commercial ELISA kits [22]. The blood pressures of all participants were measured just before taking blood samples.

Sera of patients with schizophrenia and control groups, APLN (Human APLN, catalogue no: EH2174 Fine Biotech Co., Ltd., Wuhan, China), ELA (Human Elabela, catalogue no: S1508, Peninsula Laboratories International, Inc., San Carlos, USA) and NO levels, (Human Nitric oxide, catalogue no: 201-12-1511 Sunred Biological Technology Co., Ltd., Shanghai, China) were examined according to manufacturer instructions provided in each kit.

APLN, ELA and NO measurement ranges were 62.5-4,000 pg/mL, 0-100,000 pg/mL and 4-600 mmol/L, respectively. While APLN and NO sensitivities were 37.5 pg/mL, 2.052 μmol/L respectively, intra-Assay coefficient of variation (CV) and inter-Assay CV values were < 10 and < 8, and < 12 and < 10% respectively. The average IC 50 of the ELA ELISA kit was 2.0 ng/mL. The unit of NO was kept at micromol/Liter throughout this study. However, micromole/Liter unit was converted into pg/mL to make the unit the same for all parameters used in the receiver-operating characteristics (ROC) analysis.

The Bio-Tek ELX50 (BioTek Instruments, USA) automatic plate-washer was used for plate washes, and ChroMate and Microplate Reader P4300 devices (Awareness Technology Instruments, USA) were used for absorbance readings.

### Statistical analysis

Data were analysed by SPSS version 22 software (SPSS Inc., Chicago, IL, USA). Normality of distribution was checked by visual (histograms/probability plots) and analytical (Kolmogorov-Smirnov/Shapiro-Wilk's tests) techniques. In the comparison of groups, Student's t-test was used if variables were normally distributed, whereas the Mann-Whitney U test was used if variables were not normally distributed. Categorical variables were analysed using the Chi-squared test.

Sensitivity and specificity values of APLN, ELA and NO levels for predicting schizophrenia were estimated using receiver operator characteristic curve analysis. The cut-off levels of ELA APLN, ELA/NO were determined using MedCalc 9.2.0.1 (MedCalc software, Mariakerke, Belgium). ROC curve analysis was used to measure the capacity of showing schizophrenia disease of serum ELA/APLN, ELA/NO and APLN/NO ratios. When a significant value was obtained in ROC curve analysis, sensitivity, specificity, positive predictive value and negative predictive values were calculated. *p* < 0.05 were considered statistically significant differences.

## Results

The mean age of the 33 patients (9 females, 24 males) in the study and the mean age of the 32 healthy participants (10 females, 22 males) are given in Table 1. There was no significant difference between the groups in terms of age and gender (Table 1). Demographic data and laboratory data of the groups are also given in Table 1. There was no significant difference between serum glucose and TG levels, whereas the differences between TC, LDL-C and HDL-C levels were significant. Although there was no significant difference between groups in terms of BMI and diastolic blood pressures (DBP), systolic blood pressures (SBP) were significantly different.

**Table 1:** Comparison of demographic data and laboratory findings of groups Data are shown as mean ± standard deviation. BMI, body mass index; DBP, diastolic blood pressures; FG, fasting glucose; HDL-C, high-density lipoprotein cholesterol; LDL-C, low-density lipoprotein cholesterol; SBP, systolic blood pressures; TG, triglyceride; TC, total cholesterol; p value is significant at <0.05.

	Control	Schizophrenia	p value
Number of subjects (Men/Women)	32 (22/10)	33 (24/9)	0.724
Age (year)	43.59 ± 9.22	42.51 ±11.34	0.927
Illness duration (year)	–	17.55 ± 1.99	< 0.001
BMI (kg/m^2^)	26.68 ± 3.17	25.65 ± 3.88	0.111
FG (mmol/L)	5.54 ± 0.77	5.66 ± 0.90	0.808
TC (mmol/L)	4.63± 0.60	4.04 ± 0.28	0.033
TG (mmol/L)	1.77 ± 0.10	1.87 ± 0.04	0.927
LDL-C (mmol/L)	2.08 ± 0.21	2.65 ± 0.23	0.027
HDL-C (mmol/L)	1.67 ± 0.14	1.21 ± 0.15	0.017
SBP (mmHg)	113.13 ± 17.80	117.58 ± 16.13	0.034
DBP (mmHg)	70.63 ± 9.14	73.94 ± 7.47	0.176

**Figure 1:**
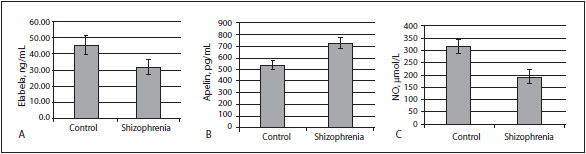
Comparison of ELA, APLN and NO between groups

The levels of ELA and NO of patients with schizophrenia were lower than the levels of ELA and NO in controls, while APLN levels were higher in schizophrenia (Figure 1). The difference between ELA, NO and APLN levels in the groups was significant (Figure 1). According to the correlation analysis, while there was a negative and significant correlation between APLN, TG and BMI levels (Table 2). A positive and significant correlation was found between ELA and BMI (Table 2).

**Table 2:** Correlation analysis APLN, apelin; BMI, body mass index; ELA, elabela; TG, triglyceride

Groups	*r* value	*p* value
TG – APLN	- 0.426	0.000
BMI – ELA	+ 0.418	0.001
BMI – APLN	- 0.330	0.007

**Figure 2:**
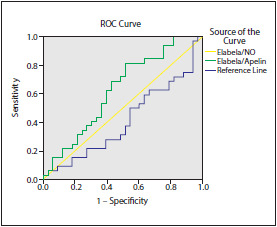
ROC analysis of elabela/NO and elabela/apelin ratios in schizophrenia patients

The results of ROC analyses to determine whether these parameters could be used for schizophrenia diagnosis were as follows: the area under curve (AUC) for the ratio of ELA/APLN was 0.628 and 95% confidence interval (CI): 0.491-0.764, whereas the AUC for ELA/NO ratio was 0.410, 95% CI: 0.270-0.550 (Figure 2).

**Figure 3:**
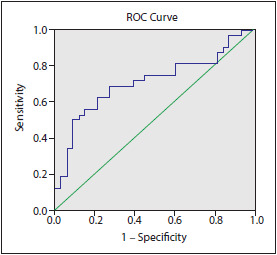
ROC analysis of apelin/NO ratio in schizophrenia patients

The AUC of the APLN/NO ratio was 0.709, 95% CI: 0.579-0.840 (p = 0.004; Figure 3). A 1.7 cut-off for APLN/NO ratio had 84% (95% CI 68-94%) sensitivity and 56% (95% CI 37-73%) specificity for a schizophrenia diagnosis. The positive predictive value of the test was also 66% and had a negative predictive value of 78%.

## Discussion

ELA levels measured in the blood of patients with schizophrenia were shown or the first time to be lower than in controls. Development of schizophrenia begins with deterioration of the development of some regions of the brain in the early neurodevelopmental process due to the effect of impaired cell signalling mechanisms in the embryonic period. It continues after the emergence of schizophrenic symptoms and is finalised through the adverse contributions of environmental, genetic and developmental factors [23] [24] [25]. Considering developmental processes, it appears that ELA is important in the meso-endodermal cell signalling activity in the embryonic period [26] [27].

The low levels of ELA in the serum of patients with schizophrenia may be associated with disorders in ELA synthesis or release during the early neurodevelopmental process. If this is the case, then we may assume that ELA may initiate schizophrenia-causing defects during the embryonic period; thus, interventions at this early stage of the disorder may be instrumental in preventing its development.

We have found no other reports of any relationship between schizophrenia and ELA. However, ELA immunoreactivity is increased with higher grade gliomas, a type of central nervous system tumour [28]. This relationship means that the ELA/APJ system is involved not only in the cardiovascular system but in the central nervous system. Besides, we have found that the APLN levels of patients with schizophrenia increased whereas NO levels remained lower than controls.

The increase of APLN in schizophrenia patients may be due to activation of a physiological protective compensatory mechanism aimed at reducing disease activity. This hypothesis is supported by Li et al. [13], who reported that intracerebrovascular injection of APLN to stressed rats induced antidepressant-like effect and activated the healing of recognition through upregulation of the PI3K and ERK1/2 signalling pathways.

However, it remains unclear why NO levels decrease even though APLN levels increase in patient serum. In a previous study, measurement of NO and NO metabolites was not found to be a proper diagnostic method for schizophrenia [29]. Considering the NO values obtained in patients with schizophrenia, we agree with Bernstein et al. [29] that NO values alone are weak in determining any characteristic of patients [29] and our results with NO may be attributed to this limitation.

There have yet to be any studies showing the reference values of APLN and NO ratio. Our study demonstrates that the mean reference value for APLN and NO ratio are 520 ± 47 and 178 ± 21 respectively. When evaluating the data in the form of APLN/NO ratio, this ratio may have diagnostic value for schizophrenia. Increased serum APLN levels may be an independent predictor of depression and anxiety development in patients having peritoneal dialysis [30]. Disorders in these parameters may be the result of schizophrenia itself, and also due to the antipsychotic agents used in treatment.

Note that our patients with chronic schizophrenia received antipsychotic treatment. Therefore, we cannot determine how serum levels of these peptides are affected by medical treatment of schizophrenia; any possible relationships may have interfered with our results. If this study was carried out with newly diagnosed schizophrenic patients who had not taken medication, the diagnostic value of APLN/NO ratio and the relationship between schizophrenia pathogenesis, and the levels of ELA, APLN and NO, would probably have been better understood.

In conclusion, this novel study investigated the relationship between schizophrenia and ELA and APLN peptides. Diagnosis of schizophrenia is currently based on physical examination, tests and screenings (such as an MRI or CT scan), psychiatric evaluation or using the criteria in the Diagnostic and Statistical Manual of Mental Disorders (DSM-5), published by the American Psychiatric Association. These clinical symptoms and laboratory results are not specific enough to distinguish schizophrenia from a number of other conditions (such as a paraphrenia). Therefore, fast and accurate diagnosis of schizophrenia is important to be able to treat this disorder. Here we reported that the APLN/NO ratio may have some use in the diagnosis of the disease. Given the increased effect of apelin on locomotor activity in animals [31], apelin may also associate with a vulnerability for schizophrenia with its direct effects on apelin receptors. NO is also involved in storage, uptake andrelease of mediators and neurotransmitters, including glutamate, acetylcholine, noradrenaline, GABA, taurine and glycine. Given the roles of NO in central nervous system development, these changes may result in neurodevelopmental changes associated with schizophrenia [24]. In general, as for the practical aspects of using this ratio in clinical and laboratory practice and comparing advantages and disadvantages concerning widely accepted procedures for diagnosis of schizophrenia, we emphasised that APLN/NO ratio calculation presents a new and amenable way for early diagnosis of schizophrenia.

Furthermore, hormones of the endocrine system may be a therapeutic target for the correction of symptoms in severe mental diseases such as schizophrenia; and some drugs that affect the hypothalamic-pituitary-adrenal axis seem to improve cognition in psychiatric diseases [32]. Therefore, in the light of our findings, this study may be helpful for the establishment of thresholds which predict disease progression in various clinical practices. However, it is clear that both clinical and experimental studies are needed to fully understand and effectively treat schizophrenia, a disease in which immunological, genetic, developmental and environmental factors are involved in etiopathogenesis.

## Conflict of interest statement

The authors state that they have no conflicts of interest regarding the publication of this article.

## List of abbreviations

APJ, apelin receptor; APLN, apelin; AUC, area under curve; BMI, body mass index; CI, confidence interval; CV, coefficient of variation; DBP, diastolic blood pressures;ELA, elabela; ERK1/2, extracellular signal-regulated kinases 1/2; HDL-C, high-density lipoprotein cholesterol; LDL-C, lowdensity lipoprotein cholesterol; NO, nitric oxide; NOS, nitricoxide synthase; PI3K, phosphatidylinositol 3-kinase; ROC, receiver-operating characteristic; SBP, systolic blood pressures; SCID, Structured Clinical Interview for DSM-IV; TC, totalcholesterol; TG, triglyceride.
